# Lithium Disilicate Ceramic Endocrown Biomechanical Response According to Different Pulp Chamber Extension Angles and Filling Materials

**DOI:** 10.3390/ma14051307

**Published:** 2021-03-09

**Authors:** João Paulo Mendes Tribst, Roberto Lo Giudice, Alison Flavio Campos dos Santos, Alexandre Luiz Souto Borges, Laís Regiane Silva-Concílio, Marina Amaral, Giuseppe Lo Giudice

**Affiliations:** 1 Postgraduate Program, School of Dentistry, University of Taubaté, Taubaté, São Paulo 12020-340, Brazil; joao.tribst@gmail.com (J.P.M.T.); flaviosantosdr@outlook.com (A.F.C.d.S.); regiane1@yahoo.com (L.R.S.-C.); marinamaral_85@yahoo.com.br (M.A.); 2 Department of Human Pathology in Adulthood and Childhood “G. Barresi”, University Hospital “G. Martino” of Messina, Via Consolare Valeria 1, 98123 Messina, Italy; 3 Institute of Science and Technology, São Paulo State University (Unesp), São José dos Campos, São Paulo 12220-690, Brazil; alexanborges@gmail.com; 4 Department of Biomedical and Dental Sciences and Morphofunctional Imaging, University of Messina, 98123 Messina, Italy; logiudiceg@unime.it

**Keywords:** dental restoration failure, endodontically treated teeth, finite element analysis, dental materials

## Abstract

The purpose of this study is to evaluate the effect of pulp chamber extension angles and filling material mechanical properties on the biomechanical response of a ceramic endocrown. A 3D model of maxillary molar that underwent endodontically treatment was exported to computer aided design software to conduct finite element analysis (FEA). The endocrown model was modified considering different pulp chamber extension angles (right angle; 6°, 12° and 18° of axial divergence). The solids were imported into the computer aided engineering software in Standard for the Exchange of Product Data (STEP) format. Nine different filling materials were simulated to seal the orifice of the root canal system under each endocrown restoration (resin composite, bulk-fill resin composite, alkasite, flowable resin composite, glass ionomer cement, autocured resin-reinforced glass ionomer cement, resin cement, bulk-fill flowable resin composite, zinc oxide cement), totaling 36 models. An axial load (300 N) was applied at the occlusal surface. Results were determined by colorimetric graphs of von-Misses stress (VMS) and Maximum Principal Stress (MPS) on tooth, cement layer, and endocrown restorations. VMS distribution showed a similar pattern between the models, with more stress at the load region for the right-angled endocrowns. The MPS showed that the endocrown intaglio surface and cement layer showed different mechanical responses with different filing materials and pulp chamber angles. The stress peaks plotted in the dispersion plot showed that the filling material stiffness is proportional to the stress magnitude in the endocrown, cement layer and tooth adhesive surface. In addition, the higher the pulp chamber preparation angle, the higher the stress peak in the restoration and tooth, and the lower the stress in the cement layer. Therefore, 6° and 12° pulp chamber angles showed more promising balance between the stresses of the adhesive interface structures. Under the conditions of this study, rigid filling materials were avoided to seal the orifice of root canal system when an endocrown restoration was planned as rehabilitation. In addition, the pulp chamber axial walls were prepared between 6° and 12° of divergence to balance the stress magnitude in the adhesive interface for this treatment modality.

## 1. Introduction

Endocrown restorations have been reported as a promising treatments to rehabilitate extensively damaged endodontically treated teeth [1,2]. The endocrown can be defined as a single piece restoration that replaces part of the crown and contains an extension to the pulp chamber. This macroretention provided by the pulp chamber axial walls associated with the adhesive luting procedure make the endocrown restoration suitable for teeth with short and/or curved roots, when the endodontic post cannot be used or when a more conservative approach is planned [1,2,3]. These different indications are based on the high success rate, higher than 91%, of this treatment modality reported for extensively damaged endodontically treated posterior teeth [4].

Endocrown restorations are indicated for posterior regions, particularly in molars with reduced clinically crowns, calcified root canals or very slender roots [5]. However, the endocrown treatment is contraindicated for substrates with insufficient adhesion, pulp chambers less than 3 mm deep or cervical margins less than 2 mm wide for most of its finishing line [5].

Part of the endocrown’s success is based on the central retainer design, since during the chewing load incidence it can act concentrating part of the stress at the pulp chamber floor, assisting the restoration and surrounding tooth structure to withstand the physiological loading [6]. The literature reports that the central retainer must follow the anatomical shape of the pulp chamber [7] and that its height in the pulp chamber must not significantly influence the fracture resistance of endocrown restorations [8]. In addition, modifying the central retainer with a different design can affect the stress distribution and fracture patterns of ceramic molar endocrowns [9].

The stress distribution pattern of a tooth that is repaired with an endocrown is vital for damage and fracture analysis [10]. Several parameters can modify the endocrown’s mechanical behavior—e.g., the ferrule presence, pulp chamber extension height and different restorative materials [11,12,13]. 

In the literature, different angles are reported during the pulp chamber preparation prior to the endocrown manufacturing. A clinical report used a cylindrical–conical diamond bur with convergence of 7° to perform the preparation [14], while in vitro studies have reported axial walls with 8° of divergence [13], values between 6° and 8° [15], 10° [16], and even 12° of divergence [7]. However, how the pulp chamber preparation angle can affect the endocrown mechanical response has not been elucidated yet.

Another aspect that is commonly performed during endocrown manufacturing is pulp chamber flattening with filling materials. When the pulp chamber floor is not completely flattened, the incidence of unfavorable failures can increase [17], and the preparation impression and the endocrown design can be compromised [14]. There are reports of different filling materials applied to seal the root canal and to flatten the pulp chamber, such as: resin cement [18], flowable composite resin [18,19], sculptable composite resin [20], and glass ionomer cement [21]. However, there is no study in the literature that has evaluated the effect of different biomaterials to flatten the pulp chamber and to support the endocrown restoration. Therefore, it would be valuable to understand how different restorative materials can affect the endocrown’s performance and the chewing load dissipation and if other filling materials can be used for this purpose.

Therefore, the goal of the present study was to investigate the effect of different pulp chamber preparation angles (right angle, 6°, 12° and 18°) and different filling materials (resin composite, bulk-fill resin composite, alkasite, flowable resin composite, glass ionomer cement, autocured resin-reinforced glass ionomer cement, resin cement, bulk-fill flowable resin composite, zinc oxide cement) on ceramic endocrown biomechanical behavior using finite element analysis. Thus, the null hypotheses were that (1) the pulp chamber preparation angle and (2) the filling material would not negatively influence the endocrown’s mechanical response and the values of maximum stress. 

## 2. Materials and Methods

**Figure 1:**
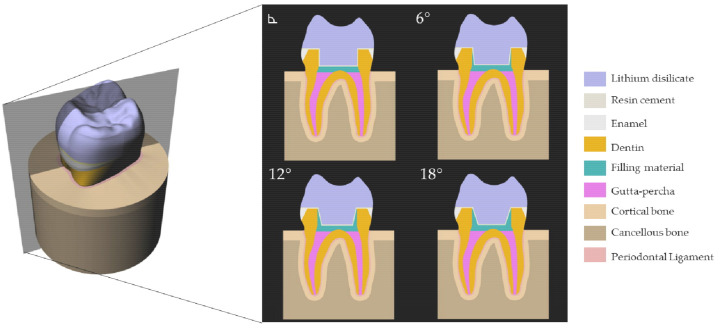
Three-dimensional model created in the modeling software with different provisional endocrown restoration.

A previous reported tridimensional first maxillary molar model was selected [22]. The Standard for the Exchange of Product Data (STEP) file was exported to the modeling software (Rhinoceros version 5.0 SR8, McNeel North America, Seattle, WA, USA). The model was composed of the following geometries: endocrown restoration, cement layer (100 µm thickness), dentin tissue, enamel tissue, periodontal ligament, cortical bone, and cancellous bone. The intaglio surface of the restoration was modified to allow a similar shape and number of the contacting faces between the endocrown and cement layer to reduce the interference during the processing [3]. In addition, a finishing line of 1 mm thickness has been considered. The endocrown restoration was considered with a thickness of 7 mm at the center of the restoration and 1.5 mm of sound enamel [22]. The model has been replicated in four different models according to the central retainer angulation (right angle, 6°, 12° and 18°). In addition, each model received a filling layer (1 mm thickness) under the endocrown restoration as the flattening procedure to seal the root canal system. Figure 1 exemplifies the different models geometries evaluated in the present study.

After the modeling process, each geometry was established as a volumetric solid without duplicate or inconsistent faces. All models were exported to the analysis software (ANSYS 19.2, ANSYS Inc., Houston, TX, USA) and a 10% convergence test of mesh control was performed to determine the number of nodes and tetrahedral elements for each endocrown model.

For the meshing, the convergence test was based on the number of nodes (196,716) and elements (94,896). The element used in the mesh division was the tetrahedral with 10 nodes (Tet-10). The mesh quality parameters were: element quality defined as 0.71 ± 0.15, aspect ratio of 1.71 ± 0.69, average maximum corner angle of 92.20° and skewness average of 0.28 ± 0.11. The inflation option was defined as a smooth transition between the geometries. The rigid body behavior has been standardized as dimensionally reduced.

According to previous reported elastic modulus and Poisson ratio, nine different filling biomaterials were simulated (resin composite, bulk-fill resin composite, alkasite, flowable resin composite, glass ionomer cement, autocured resin-reinforced glass ionomer cement, resin cement, bulk-fill flowable resin composite, zinc oxide cement). The mechanical properties of each material/structure (Table 1) were inserted into the analysis software and each material was considered as isotropic and homogeneous [23,24,25,26,27,28,29,30,31,32]. 

Regardless of the filling material and central retainer angulation, the endocrown material was standardized as lithium disilicate based ceramic. The test was performed considering a condition of no-failure with elastic materials. Ideal contacts were considered between the volumes. In the boundary condition, the fixation was applied at the base of the bone tissue and fixed with zero nodal displacement. The occlusal loading (300 N) was applied to simulate a compressive load in the center of the crown [22]. Von-Mises stress was recorded as a general view of the biomechanical behavior and for the restoration, cement layer and tooth adhesive surface, the maximum principal stress (in MPa) criteria were adopted as failure criteria. In the postprocessing step, qualitative stress maps were generated from the software, and the highest peak in each structure was calculated. A different stress peak higher than 10% was assumed as a relevant comparison. The linear proportion between stress peaks and filling material elastic moduli was calculated as dispersion plot for each analyzed structure (endocrown, cement and tooth). 

## 3. Results

**Figure 2:**
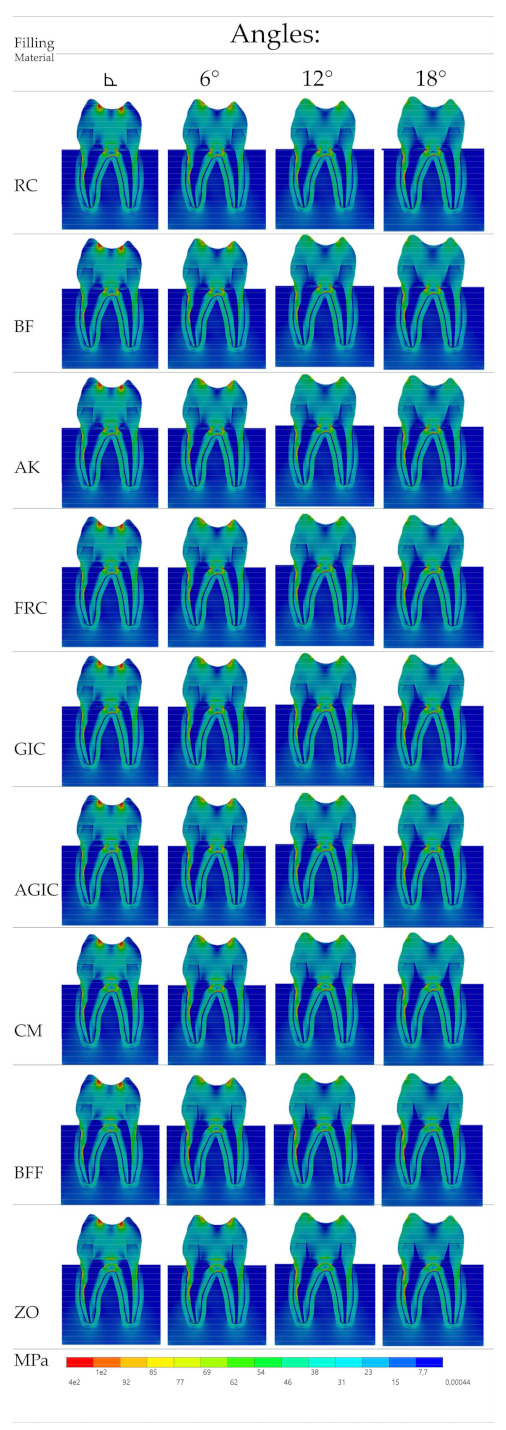
Von-Misses stress distribution in the endocrown section plane according to the pulp chamber axial walls angle (columns) and restorative material (rows). The corresponding filling materials are: resin composite, bulk-fill resin composite, alkasite, flowable resin composite, glass ionomer cement, autocured resin-reinforced glass ionomer cement, resin cement, bulk-fill flowable resin composite, and zinc oxide cement.

**Figure 3:**
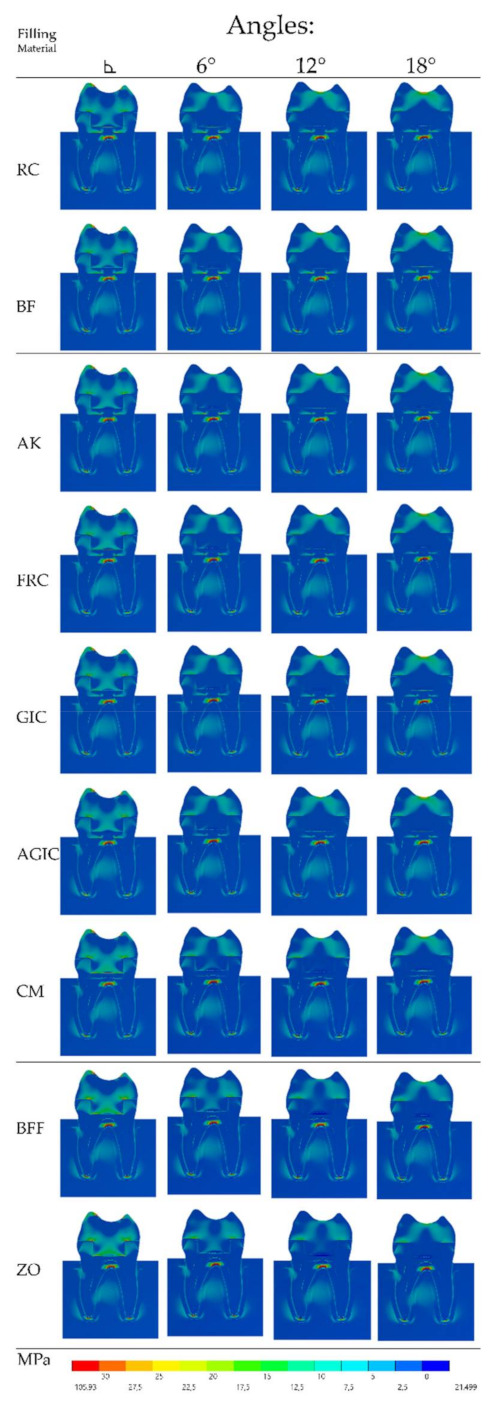
Tensile stress distribution in the endocrown section plane according to the pulp chamber axial walls angle (columns) and restorative material (rows). The corresponding filling materials are: resin composite, bulk-fill resin composite, alkasite, flowable resin composite, glass ionomer cement, autocured resin-reinforced glass ionomer cement, resin cement, bulk-fill flowable resin composite, and zinc oxide cement.

After the processing, Von-Mises (VMS) and Maximum Principal Stress (MPS) results (MPa) were obtained for the models. The stress data were summarized using colorimetric maps (Figure 2 and Figure 3). For the Von-Mises stress (Figure 2), a similar stress trend was calculated regardless the filing material and central retainer angle; however, the less rigid the filling material was, the lower the stress concentration in the endocrown occlusal surface near the load application area. In addition, for the central retainer (in the pulp chamber region) near the filling material, different stress magnitude can be noticed with more presence of green and yellow fringes when rigid filling materials were considered. 

**Figure 4:**
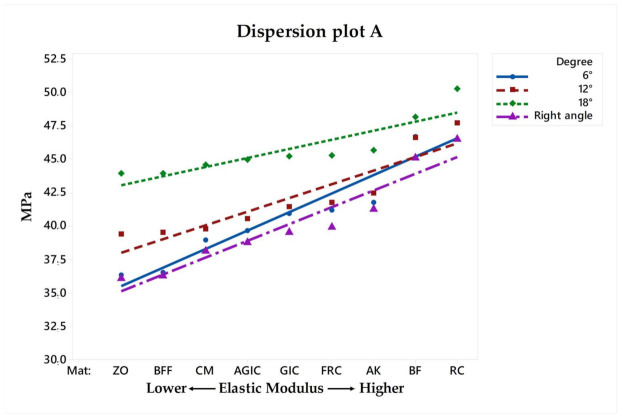
Dispersion plot according to the filling material elastic moduli and stress peaks recorded in the endocrown restoration.

**Figure 5:**
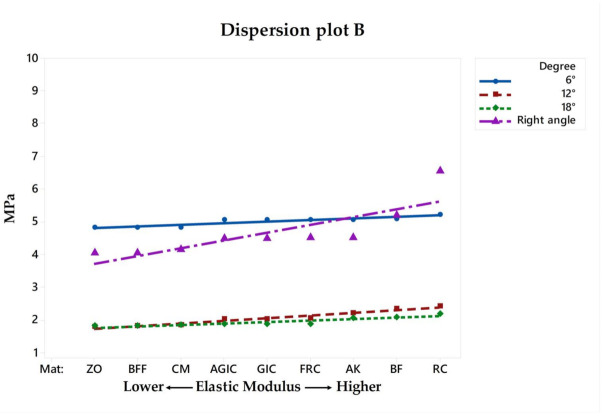
Dispersion plot according to the filling material elastic moduli and stress peaks recorded on the cement layer.

**Figure 6:**
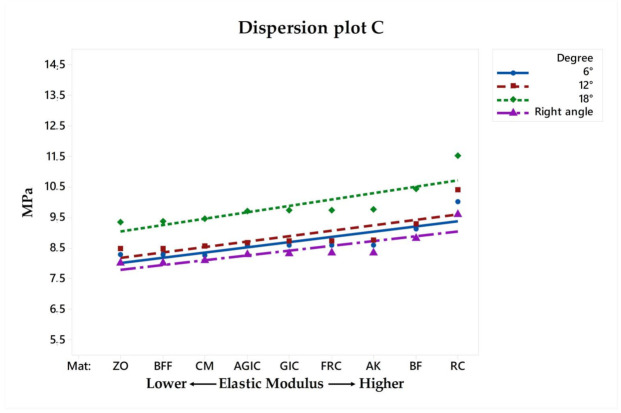
Dispersion plot according to the filling material elastic moduli and stress peaks recorded in the tooth adhesive area.

Similar to the VMS, the MPS (Figure 3) stress maps showed a proportional pattern of distribution between the structures as the filling material elastic modulus increased. There was a high stress magnitude at the fulcrum between the roots; however, this was the case in all models regardless the endocrown design and filling material. Focusing on the restoration failure, three different stress values were recorded for each model at the restoration’s intaglio surface, cement layer, and adhesive area of the pulp chamber. The stress peaks were plotted in a distribution plot according to the MPa values and filling materials that were simulated (Figure 4, Figure 5 and Figure 6).

For the restoration, regarding the different pulp chamber axial walls angulations, the stress peaks ranged from 36.09 to 46.44 in right-angled endocrowns, from 36.33 to 47.66 when 6° of divergence was simulated, from 39.35 to 47.70 when 12° of divergence was simulated, and, from 43.88 to 50.23 when 18° of divergence was simulated. These differences in stress values were proportional to the filling material stiffness and pulp chamber axial walls preparation, as can be observed in Figure 4 and Figure 6. Therefore, the higher the elastic modulus under restoration and preparation angle, the higher the stress magnitude at the central retainer of the endocrown. A similar effect can be observed for the stress in the pulp chamber dentin tissue, showing that the composite resin as the filling material in the 18° preparation can increase the stress at the tooth adhesive surface.

Another structure that was separately evaluated was the cement layer, which is responsible for the adhesion between the ceramic material and the tooth substrate. Opposite to what happened in the ceramic and dentin, the cement layer stress magnitude was inversely proportional to the filling material elastic modulus and pulp chamber preparation angle (Figure 5). In this sense, the models that are positive for the endocrown’s stress reduction can behave negatively for the cement layer, increasing stress magnitude. Therefore, the total amount of stress in the adhesive interface (restoration, cement and substrate) should be considered prior to the indication of the most promising combination of clinical parameters.

## 4. Discussion

The null hypotheses were that the pulp chamber preparation angle and the different filling materials would not negatively influence the mechanical response and the values of maximum stress. The results showed that both factors influenced the biomechanical response of the ceramic endocrown restoration. Thus, the hypotheses of the present study were rejected. It was observed that the filling material with a high elastic modulus (RC, BF, AK, FRC) allowed less stress magnitude to reach the cement layer; however, it increased the stress magnitude in the tooth substrate as well as in the restoration. In addition, the literature reports that more flexible restorative materials could be a promising option for the promotion of adequate biomechanical behavior for the endocrown restorations [3,4,6,15,18,22]. This study complements this finding, showing that the filling material stiffness can also modify the stress concentration in the restoration. 

The use of a ceramic endocrown is indicated for a long-term period; therefore, the restoration longevity must be considered in order to ensure the clinical success of the restoration without catastrophic or adhesive failures that can affect the patient’s life [1,4]. Therefore, results suggesting higher risk for restoration debonding or marginal infiltration can be evaluated considering the cement layer stress magnitude [3,6,7]. 

According to a review [20] that evaluated the clinical behavior of endocrown restorations, the calculated success rate for molars ranged between 72.73% and 99.57%. In addition, the most common mode of failure was the restoration debonding [20]. From the present results, use of less rigid filling materials—e.g., bulk-fill flowable resin—can offer the patient a more reliable treatment option in terms of stress and adhesive failure risk. It is important to note that the endocrown is a treatment modality that can promote high tensile stress magnitude at the cement layer and in the substrate [33], presenting a fracture pattern that can be influenced by the restorative material property of integrated crack prevention [34,35]. 

The magnitude of generated stress on the cement layer, in the substrate and restorative material, is usually proportional to the debonding failure risk [8]. In addition, the endocrown internal adaptation can affect the cement layer thickness [11,35] and consequently the polymerization shrinkage effect and defects on its structure. However, in this investigation, all models were simulated with a uniform cement thickness, which cannot occur clinically [33]. 

In a 10-year retrospective study, from 99 endocrowns, two cases of caries recurrence were reported in the restoration margin [35]. As can be seen in Figure 4, the higher the elastic modulus, the higher the stress magnitude. This effect can be even higher when higher degree of pulp chamber preparation was performed during the restoration plan. 

In addition to the evaluated materials, the chewing incidence can also affect the mechanical response of the endocrown treatment [11,33]. In this study, the axial load was applied in order to simulate the most common load (axial) in the posterior region [11,18,22]. However, the nonaxial chewing load incidence and inclined planes of cusp heights [36,37,38] can negatively affect the endocrown mechanical response. This study corroborates this mechanical behavior, since the stress concentration is very similar between the groups (Figure 2andFigure 3). However, during the endocrown preparation, increasing the pulp chamber axial walls angle can improve the restoration behavior. It is important to note that the use of a flat pulp chamber floor can increase the load to fracture [7,17]. To complement this, the present study suggests that an easy applicable biomaterial should be used during the flattening step; however, a more flexible material should always be preferred and considered during the treatment plan.

A previous study reported that the central retainer is necessary to promote an adequate load dissipation in this restoration modality [7]. Therefore, smoothening the edges at the central retainer in endocrown preparation is suggested to reduce the stress magnitude in the dentin tissue and the cement layer [7]. The present results demonstrate that the filling material used during the flattening preparation should be chosen to avoid higher stress concentration at the adhesive interface. 

According to the literature [3,6,22,33,34], the lower the stiffness of the endocrown restorative material, the higher the calculated stresses in the tooth tissues and at the adhesive interface between the ceramic and cement. This pattern has been observed in the present results, corroborating to indicate a more flexible filling material to seal the root canal system.

The clinically recommended angle of preparation for a complete crown is between 4° and 14°, considering the retention of restoration and convenience of simultaneous insertion [7].

Despite the literature reporting endocrown preparations with different central retainer angulation, ranging from 6° to 12° of divergence [7,13,14,15,16], there is no mechanical reason for the selected angle parameter. Apparently, a citation chain occurred between the previous reports and there was no assessment regarding how the preparation angle can affect the restoration load dissipation.

A previous study [7] applied finite element analysis to determine the effect of the central retainer shape and abduction angle of the abutment on the stress distribution in the cervical dentin and cement layer. The authors concluded that the angle formed by the opposing lateral dentin walls would not affect the endocrown’s mechanical behavior. Opposite to this, the present study showed that there is a difference between the axial wall’s preparation angles at the pulp chamber floor stress. Comparing both studies, the major difference in boundary conditions was the load incidence, since the previous study [6] (45°-angled load) and the present study applied axial chewing loads. In addition, the present study considered cortical and cancellous bone tissue as separated structures and just 1.5 mm of enamel height, considered as the minimum necessary for endocrown manufacturing [18].

With regards to the flattening of the pulp chamber floor, previous studies reported different filling materials, such as: resin cement [18], flowable composite resin [19], moldable composite resin [20], and glass ionomer cement [21]. However, the reason to choose the filling material used to flatten the pulp chamber restoration has never been justified before. Ideally, the materials should be adhesive and allow an easy application, remain in position during the cavity preparation refinement, and should be chemically compatible with the resin cement. Since the present study demonstrates that a more flexible material can reduce the stress concentration at the adhesive interface, it is possible to suggest the bulk-fill flowable resin composite and the resin cement for this indication. The zinc oxide cement was the most flexible one; however, the presence of eugenol can negatively affect the polymerization shrinkage of resin cement [39] and also it is not as adhesive as the other resinous filling materials.

In the case of resin composite filling, the surface roughness can affect the hardness of the material; additionally, the responses to the dynamic loading are not identical due to the different compositions and matrix ratios of different restorative resin composites [39]. Therefore, it is important to consider the mechanical properties and failure modes of the resin composite during the treatment plan [40]. In addition, the application method can also be a determinant factor for the success of restoration. The literature reports that conventional incrementally placed composite has a higher degree of conversion compared to bulk-fill materials, and that the bulk-fill material presents a microleakage potential similar to the conventional resin composite [41]. The present study complements these findings, suggesting that the bulk-fill and bulk-fill flowable composites are more interesting options to be used as filling materials in endocrown treatment, associating the reported literature benefits [40,41] with reduced stress concentration at the adhesive interface when compared with conventional resin composite.

In the present study, the design criteria of the filling material layer were used based on previous in vitro [1,2,15,25] and in vivo [14,20] reports. Therefore, the concept is to fill the pulp chamber with the restorative material and then, to perform an adequate endocrown preparation [18]. In the modeling step, this design was performed using a Boolean difference between the endocrown pulp chamber extension and the filling material followed by the cement layer modeling using offset surfaces from the restoration intaglio-surface. At the end of this process, the cement layer and filling material presented homogenous thickness, an ideal situation that cannot always occur clinically.

The literature suggests that an endocrown is a conservative dental treatment with adequate longevity for posterior teeth with endodontic treatment [1,4,33,42]. The present study corroborates this statement, demonstrating low stress magnitude regardless of the preparation angle and filling material of the pulp chamber axial walls. However, when performing an in silico simulation, some methodological limitations should be considered. In the oral cavity, the restoration would suffer pH and temperature variations and different chewing loads incidences. In addition, the simulated materials were considered isotropic without any incorporation of defects. The restoration fitting was considered perfect and the cement thickness homogeneous [33]. Although the FEA provides general information about the mechanical behavior of determined models, clinical results may not totally replicate the FEA findings due to the presence of simplifications and biological aspects that are not simulated. In this sense, further studies should be carried out to elucidate the endocrown load to fracture, fatigue life, microleakage, and the internal fit when different filling materials are used in the flattening step of the preparation. 

## 5. Conclusions

Within the limitations of this study, flexible filling materials, e.g., resin cement and bulk-fill flowable resin composite, should be preferred to seal the orifice of the root canal system when an endocrown restoration is planned as rehabilitation. In addition, the pulp chamber axial walls should be prepared using 6° and 12° divergences to balance the stress magnitude in the adhesive interface for restorative modality.

## Figures and Tables

**Table 1:** Elastic moduli ^1^ of the materials used in this study.

Abbreviation	Materials/Structures	Elastic Modulus (GPa)	Poisson Ratio	References
-	Lithium disilicate	95	0.30	[23]
-	Enamel Tissue	70	0.30	[23]
-	Dentin Tissue	18	0.30	[23]
-	Cancellous bone	1.37	0.30	[24]
-	Cortical bone	13.7	0.30	[24]
-	Periodontal Ligament	0.05	0.45	[25]
RC	Resin composite	13.45	0.17	[26]
BF	Bulk-fill resin composite	13.46	0.18	[26]
AK	Alkasite	13.00	0.30	[28]
FRC	Flowable resin composite	8.0	0.20	[27]
GIC	Glass Ionomer Cement	8.0	0.25	[29]
AGIC	Autocured resin-reinforcedGlass ionomer cement	8.32	0.27	[30]
CM	Resin Cement	8.6	0.18	[23]
BFF	Bulk-fill flowable resin composite	3.70	0.30	[31]
ZO	Zinc oxide cement	1.35	0.30	[32]

^1^ Values obtained in the literature.

## Data Availability

The data presented in this study are available on request from the corresponding author.
